# Previous Solid Organ Transplantation Influences Both Cancer Treatment and Survival Among Colorectal Cancer Patients

**DOI:** 10.3389/ti.2024.13173

**Published:** 2024-09-20

**Authors:** Henrik Benoni, Caroline Nordenvall, Vivan Hellström, Caroline E. Dietrich, Anna Martling, Karin E. Smedby, Sandra Eloranta

**Affiliations:** ^1^ Department of Surgical Sciences, Section of Transplantation Surgery, Uppsala University, Uppsala, Sweden; ^2^ Division of Clinical Epidemiology, Department of Medicine, Solna, Karolinska Institutet (KI), Stockholm, Sweden; ^3^ Department of Molecular Medicine and Surgery, Karolinska Institutet (KI), Stockholm, Sweden

**Keywords:** cancer after transplant, colorectal cancer (CRC), organ transplantation, survival, mortality

## Abstract

Previous solid organ transplantation has been associated with worse survival among colorectal cancer (CRC) patients. This study investigates the contribution of CRC characteristics and treatment-related factors to the differential survival. Using the Swedish register-linkage CRCBaSe, all patients with solid organ transplantation before CRC diagnosis were identified and matched with non-transplanted CRC patients. Associations between transplantation history and clinical CRC factors and survival were estimated using the Kaplan-Meier estimator and logistic, multinomial, and Cox regression, respectively. Ninety-eight transplanted and 474 non-transplanted CRC patients were followed for 5 years after diagnosis. Among patients with stage I-III cancer, transplanted patients had lower odds of treatment with abdominal surgery [odds ratio (OR):0.27, 95% confidence interval (CI):0.08–0.90], than non-transplanted patients. Among those treated with surgery, transplanted colon cancer patients had lower odds of receiving adjuvant chemotherapy (OR:0.31, 95% CI:0.11–0.85), and transplanted rectal cancer patients had higher rate of relapse (hazard ratio:9.60, 95% CI:1.84–50.1), than non-transplanted patients. Five-year cancer-specific and overall survival was 56% and 35% among transplanted CRC patients, and 68% and 57% among non-transplanted. Accordingly, transplanted CRC patients were treated less intensely than non-transplanted patients, and had worse cancer-specific and overall survival. These patients might benefit from multidisciplinary evaluation including transplantation specialists.

## Graphical Abstract

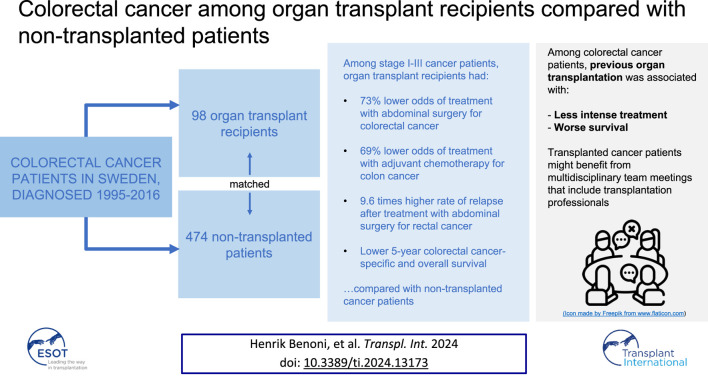

## Introduction

Solid organ transplantation is associated with both an increased risk of post-transplant cancer, and decreased overall and cancer-specific survival after cancer diagnosis, for numerous cancer types [1–5]. One such example is colorectal cancer (CRC), which has been associated with an increased rate of cancer-specific death among organ transplant recipients (OTRs), compared to CRC patients without transplantation history [4, 5]. Reasons for this difference might include variations in biological cancer characteristics (e.g., tumor grade, aggressiveness), and cancer treatment, where OTRs risk receiving limited treatment due to heavier comorbidity and/or concern for the transplanted organ. End-stage organ failure and organ transplantation are associated with systemic comorbidity to a variable extent during the post-transplant period, which may have implications for primary cancer treatment (e.g., surgery under general anesthesia). Also, neoadjuvant and adjuvant treatments such as chemotherapy or immunotherapy can have detrimental effects on the transplanted organ (e.g., nephrotoxicity), interact with immunosuppressive drugs, or induce rejection of the transplant [6]. Therefore, post-transplant cancer treatment represents a major challenge from both a clinical and scientific perspective, due to the difficulties involved with designing randomized trials of optimal treatments.

The aims of this study were to elucidate reasons for the previously shown excess rate of cancer-specific mortality among post-transplant CRC patients, through examining tumor characteristics, differences in treatment practices and relapse rates among post-transplant and non-transplanted CRC patients, and to confirm associations with survival.

## Materials and Methods

### Data Sources

This matched cohort study utilized data from CRCBaSe, a research database originating from the Swedish Colorectal Cancer Register, which includes rectal cancer diagnoses from 1995 and colon cancer diagnoses from 2007, through 2016 [7]. Reporting to this register, which has been shown to be >98% complete, is done by the treating physicians and the data contain detailed tumor and treatment information (e.g., pathological stage and tumor grade, tumor location, surgery type, and [neo]adjuvant chemo- and radiotherapy) [8]. CRCBaSe includes linkages to several population-based registers, such as the Swedish Cancer Register (SCR), the National Patient Register (NPR) (in- and outpatient specialist care visits and hospitalizations), and the Cause of Death Register. Linkage between the registers is enabled via the unique personal identity number that is assigned to all Swedish residents.

The study was approved by the Regional Ethics Review Board in Stockholm (approval no. 2007/1335-31/4, 2014/71-31 and 2016/876-32).

### Study Population

Using transplantation surgery procedure codes (Supplementary Table S1), we identified all CRC patients in CRCBaSe who had undergone any solid organ transplantation (kidney, liver, pancreas, heart, lung, bowel, and combinations thereof) prior to first CRC diagnosis (n = 121). CRC patients who were transplanted less than 30 days before their cancer diagnosis were excluded (n = 1), as were CRC patients who had a record of transplantation after their cancer diagnosis (n = 21), since they represent a distinct clinical group, and/or may have been diagnosed with cancer *en passant* around the time of transplantation surgery, in accordance with previous studies [2, 5].

A comparator group was selected, also using CRCBaSe as the source population, by random sampling (with replacement) of up to five matched, non-transplanted CRC patients to each transplanted. The matching was performed on cancer location (colon/rectum), sex, age at CRC diagnosis (±1 year), and year of diagnosis. The first recorded diagnosis of CRC (if more than one was available in CRCBaSe) in each patient was used for matching. Further, patients with synchronous tumors were excluded (1 transplanted patient [together with the corresponding 5 comparators] and 12 comparators).

### Comorbidity and Other Potential Confounders

Organ transplantation is heavily associated with comorbidity, which can be difficult to adjust for since the comorbid conditions are often closely related to exposure (e.g., severe kidney disease and kidney transplantation). Careful consideration of how to disentangle confounding from conditions that are interrelated with organ transplantation is thus important in order not to attenuate the effect of the exposure under study. For descriptive purposes, we classified comorbidities at baseline (i.e., at CRC diagnosis) using the Charlson comorbidity index (CCI) based on records of healthcare visits from the NPR (last 5 years before diagnosis) and previous non-CRC diagnoses from the SCR (last 10 years before diagnosis) [9]. Next, to address confounding by comorbidities not directly linked to the indication for the transplantation itself, we created a composite comorbidity variable for cardiovascular and other diseases that could affect choice of surgical and/or oncological treatment (i.e., cerebrovascular disease, hemiplegia, congestive heart failure, myocardial infarction, and peripheral vascular disease). Hypertension, not included in CCI, was assessed separately using ICD-9/10 diagnosis codes (440–447, I10-I15). Furthermore, as a proxy for kidney function at CRC diagnosis, we assessed occurrence of chronic dialysis at or within 180 days before CRC diagnosis using ICD-9/10 diagnosis codes (V45B, Z49, and Z99.2) as well as procedure codes (9912, DR014, and DR016). Since the NPR only includes visits to doctors (and not nurses), the 180 days cutoff was intended to ascertain that patients on dialysis had been registered as having seen a specialist physician in that time period. Naturally, among patients on chronic dialysis, we also ascertained that no kidney transplant procedure had been performed until CRC diagnosis.

Additionally, highest attained level of education (<10 years, 10–12 years, or >12 years) was used as a proxy for socioeconomic status, and administrative region (Stockholm/Gotland, Uppsala/Örebro, South East, South, West, or North) was used to account for potential regional differences in treatment.

### Cancer Characteristics, Treatment and Outcome

We studied the association between transplantation history and patient and tumor characteristics, selected based on their clinical relevance for prognosis and treatment selection. These included cancer stage at diagnosis (I-II, III, or IV), case discussion at a pre- or postoperative multidisciplinary team (MDT) meeting (yes/no), and having undergone abdominal surgery (yes/no). For stage I-III CRC patients treated with abdominal surgery, we additionally assessed association with cancer location (right-sided [including transverse colon] or left-sided for colon cancer, and distance [<6 or 6-15 cm] from the anal verge for rectal cancer) [10], low/high tumor grade (well/moderately differentiated or poorly differentiated/undifferentiated), microscopically radical surgery (yes/no), perforations found perioperatively (yes/no), perineural invasion (yes/no), administered neoadjuvant and planned adjuvant treatment (chemo- and/or radiotherapy), number of lymph nodes examined (as a proxy for the extent of surgical dissection), and whether emergency surgery was performed (yes/no) [11, 12]. For patients with palliative disease, clinical TNM (tumor-node-metastasis) status, site of distant metastases, and planned palliative treatment (yes/no, referring to chemo- and/or radiotherapy) were included.

Other outcomes of interest were cancer-specific survival and overall survival (OS) among all patients, and relapse rates among stage I-III CRC patients treated with abdominal surgery. Deaths due to CRC (i.e., cancer-specific deaths) were identified based on recorded cause of death information and the following three conditions: 1) if the diagnosis was colon cancer and the main cause of death was colon or recto-sigmoid cancer; 2) if the diagnosis was rectal cancer and the cause of death was recto-sigmoid or rectal cancer; or 3) if CRC was the only cancer in the patient’s medical history, and the recorded cause of death was any cancer, in accordance with previous studies [5, 13]. In addition, we assessed mortality due to infection and cardiovascular disease, as well as other causes.

### Statistical Analysis

All regression models were adjusted for the matching factors as well as for healthcare region, cardiovascular comorbidity, highest attained level of education, and chronic dialysis at CRC diagnosis. Logistic regression was used to estimate odds ratios (OR) and 95% confidence intervals (CI) for the association between exposure to transplantation and the various outcomes representing cancer characteristics and treatment. For cancer stage, a non-binary outcome, multinomial regression was used to estimate relative risk ratios (RRR). Individuals with missing data were excluded from the regression analyses.

In the analyses of cancer-specific survival and OS, patients were followed from the date of first CRC diagnosis (in analyses of all patients) or surgery (in analyses of stage I-III CRC patients treated with abdominal surgery), until date of death or 31st December 2017, as cause of death information was not available after 2017. However, in the relapse rate analysis, as well as in a supplementary OS analysis including stage I-III CRC patients treated with abdominal surgery, administrative censoring was set to 4th June 2022, as information on relapse and vital status was available until this date. In all survival analyses, follow-up was restricted to the first 5 years, as both relapses and cancer-related deaths typically occur within that time frame, and relapses were not systematically registered beyond 5 years after surgery.

The Kaplan-Meier method was used to estimate 5-year cancer-specific (net) survival and OS by transplantation history, and differences were evaluated with the log-rank test. Cox proportional hazards regression was used to estimate hazard ratios (HR) for relapse, and for cancer-specific and overall mortality. Furthermore, we performed sensitivity analyses focusing on survival and relapse that were limited to kidney transplant recipients and their matched comparators. The proportional hazards assumption was evaluated with the Grambsch-Therneau test on the Schoenfeld residuals [14].

SAS version 9.4 (Copyright ^©^ 2002–2012 by SAS Institute Inc., Cary, NC, United States) and Stata version 16 (StataCorp. 2019. Stata Statistical Software: Release 16. College Station, TX: StataCorp LLC.) were used for data management and statistical analysis.

## Results

The final study cohort included 572 CRC patients diagnosed in Sweden 1995-2016 (98 OTRs, 73 with colon cancer and 25 with rectal cancer; and 474 non-transplanted, 352 with colon and 122 with rectal cancer) (Table 1). Sixty-one percent were male, and 81% were 60 years or older at CRC diagnosis. The median follow-up time was 3.1 years (interquartile range 1.2–5.0 years). Among the OTRs, 77% had undergone kidney transplantation, 16% liver transplantation, 4% heart or lung transplantation, and 3% combined pancreas and kidney transplantation. Most patients (88%) had undergone one transplantation procedure, 10% two procedures, and 2% three procedures, prior to CRC diagnosis (Table 1). The median time from first transplantation to CRC diagnosis was 12 years (range 0.6–45 years). Of OTRs, 31% had cardiovascular comorbidity (compared with 14% among non-transplanted patients), and 6% had chronic dialysis at CRC diagnosis (compared with 0.04% among non-transplanted patients) (Table 1).

**TABLE 1:** Demographic characteristics of colorectal cancer patients diagnosed in Sweden between 1995 and 2016, by medical history of solid organ transplantation. Organ transplant recipients (OTR) denotes patients with any type of solid organ transplantation prior to their first colon or rectal cancer, and *No Tx* denotes non-transplanted cancer patients.

Characteristics	All colorectal cancer		Colon cancer		Rectal cancer	
	OTRs	No Tx	OTRs	No Tx	OTRs	No Tx
	N (%)	N (%)	N (%)	N (%)	N (%)	N (%)
Total no of patients	98 (100)	474 (100)	73 (100)	352 (100)	25 (100)	122 (100)
Sex						
Male	60 (61)	289 (61)	45 (62)	214 (61)	15 (60)	75 (61)
Female	38 (39)	185 (39)	28 (38)	138 (39)	10 (40)	47 (39)
Age at diagnosis (years)						
40–59	18 (18)	89 (19)	13 (18)	62 (18)	5 (20)	27 (22)
60–69	42 (43)	211 (45)	34 (47)	169 (48)	8 (32)	42 (34)
70–89	38 (39)	174 (37)	26 (36)	121 (34)	12 (48)	53 (43)
Year of diagnosis						
1995–2006	10 (10)	48 (10)	0 (0)	0 (0)	10 (40)	48 (39)
2007–2011	42 (43)	204 (43)	36 (49)	174 (49)	6 (24)	30 (25)
2012–2016	46 (47)	222 (47)	37 (51)	178 (51)	9 (36)	44 (36)
Region						
Stockholm-Gotland	14 (14)	95 (20)	10 (14)	74 (21)	4 (16)	21 (17)
Uppsala-Örebro	24 (24)	108 (23)	19 (26)	82 (23)	5 (20)	26 (21)
South East	8 (8)	46 (10)	6 (8)	30 (9)	2 (8)	16 (13)
South	19 (19)	99 (21)	11 (15)	68 (19)	8 (32)	31 (25)
West	19 (19)	85 (18)	13 (18)	68 (19)	6 (24)	17 (14)
North	14 (14)	41 (9)	14 (19)	30 (9)	0 (0)	11 (9)
Attained level of education						
<10 years	35 (36)	145 (31)	26 (36)	105 (30)	9 (38)	40 (33)
10–12 years	37 (38)	209 (44)	26 (26)	158 (45)	11 (46)	51 (42)
>12 years	25 (26)	116 (25)	21 (29)	86 (25)	4 (17)	30 (25)
*Missing*	*1*	*4*	*0*	*3*	*1*	*1*
First transplant organ type						
Kidney	75 (77)		54 (74)		21 (84)	
Liver	16 (16)		12 (16)		4 (16)	
Heart and/or lung	4 (4)		4 (5)		0 (0)	
Pancreas and kidney	3 (3)		3 (4)		0 (0)	
Number of transplantations^a^						
1	86 (88)		63 (86)		23 (92)	
2	10 (10)		8 (11)		2 (8)	
3	2 (2)		2 (3)		0 (0)	
Cardiovascular comorbidity^b^						
Yes	30 (31)	64 (14)	23 (32)	50 (14)	7 (28)	14 (11)
No	68 (69)	410 (87)	50 (68)	302 (86)	18 (72)	108 (89)
Chronic dialysis at cancer diagnosis^c^						
Yes	6 (6)	2 (0)				
No	92 (94)	472 (100)				

Abbreviations: OTR, organ transplant recipient; Tx, transplantation; N, number.

^a^ Before first colorectal cancer diagnosis.

^b^ Composite of pre-CRC diagnosis of cerebrovascular disease, hemiplegia, congestive heart failure, myocardial infarction, and peripheral vascular disease.

^c^ All OTRs with chronic dialysis had a kidney (including kidney and other) transplant. Frequencies for colon cancer and rectal cancer patients not shown due to single observations.

### Cancer Characteristics and Treatment

Overall, the distribution of cancer stage and the odds of case discussion at a preoperative MDT meeting were not statistically different between transplanted and non-transplanted CRC patients (Table 2). However, among stage I-III CRC patients, OTRs overall had 73% lower odds (OR:0.27, 95% CI:0.08–0.90), and OTRs with colon cancer had 84% lower odds (OR:0.16, 95% CI: 0.03–0.94), of receiving treatment with abdominal surgery than non-transplanted patients (Table 2).

**TABLE 2:** Characteristics of colorectal cancer patients diagnosed 1995–2016, by medical history of solid organ transplantation. The odds ratios (OR) and relative risk ratios (RRR) compare organ transplant recipients (OTRs) to non-transplanted cancer patients (No Tx) with respect to cancer characteristics and treatment.

Characteristics	All colorectal cancer				Colon cancer				Rectal cancer			
	OTRs	No Tx	OR^a^ ^,^ ^b^ (95% CI)	p-value	OTRs	No Tx	OR^a^ ^,^ ^b^ (95% CI)	p-value	OTRs	No Tx	OR^a^ ^,^ ^b^ (95% CI)	p-value
	N (%)	N (%)			N (%)	N (%)			N (%)	N (%)	
Cancer stage				0.08				0.11				0.47
I-II	33 (40)	187 (43)	Base outcome		25 (37)	138 (40)	Base outcome		8 (50)	49 (53)	Base outcome	
III	19 (23)	134 (31)	0.81 (0.43–1.53)		15 (22)	102 (30)	0.82 (0.39–1.71)		4 (25)	32 (34)	0.90 (0.23–3.55)	
IV	31 (37)	113 (26)	1.53 (0.89–2.64)		27 (40)	101 (30)	1.68 (0.89–3.19)		4 (25)	12 (14)	2.35 (0.52–10.6)	
*Missing*	*15*	*40*			*6*	*11*			*9*	*29*		
Preoperative MDT meeting				0.64				0.77				0.59
Yes	53 (60)	276 (64)	0.88 (0.52–1.49)		40 (55)	204 (58)	0.91 (0.50–1.67)		13 (81)	72 (88)	0.65 (0.13–3.13)	
No	36 (40)	157 (36)	Base outcome		33 (45)	147 (42)	Base outcome		3 (19)	10 (12)	Base outcome	
*Missing*	*9*	*41*			*0*	*1*			*9*	*40*		
Abdominal surgery **^c^**				0.03				0.04				0.32
Yes	47 (90)	306 (96)	0.27 (0.08–0.90)		37 (93)	232 (97)	0.16 (0.03–0.94)		10 (83)	74 (93)	0.33 (0.04–2.98)	
No	5 (10)	14 (4)	Base outcome		3 (8)	8 (3)	Base outcome		2 (17)	6 (8)	Base outcome	
*Missing*	*0*	*1*			*0*	*0*			*0*	*1*		

Abbreviations: OTR, organ transplant recipient; Tx, transplantation; OR, odds ratio; CI, confidence interval; N, number; MDT, multidisciplinary team.

^a^ All reported ORs and relative risk ratios (RRR) refer to the contrast between OTRs, and non-transplanted cancer patients (No Tx), where the latter constitute the reference group. The regression models were adjusted for the matching factors cancer location (colon or rectum), sex, age at diagnosis (±1 year), and year of CRC, diagnosis, as well as region, cardiovascular comorbidity, attained level of education, and chronic dialysis at cancer diagnosis.

^b^ RRR from multinomial regression for reporting association with cancer stage.

^c^ Among stage I-III patients.

Furthermore, among stage I-III colon cancer patients treated with abdominal surgery, OTRs had higher odds of right-sided colon cancer (OR:3.74, 95% CI:1.59–8.84) compared to non-transplanted patients, and adjuvant chemotherapy was planned for 6 (19%) transplanted and 86 (43%) non-transplanted patients with stage II-III cancer (Table 3). This corresponded to OTRs having 69% lower odds of adjuvant treatment than non-transplanted CRC patients (OR:0.31, 95% CI:0.11–0.85). Six (43%) OTRs, compared with 64 (64%) non-transplanted patients, with stage III cancer were treated with adjuvant chemotherapy (OR:0.33, 95% CI:0.07–1.67) (Table 3). Among the OTRs with adjuvant treatment, 3 (50%) had right-sided and 3 (50%) left-sided colon cancer, while among the non-transplanted, 41 (48%) had right-sided and 45 (52%) had left-sided cancer.

**TABLE 3:** Characteristics of stage I-III colon cancer patients treated with abdominal surgery, by medical history of solid organ transplantation. The odds ratios (OR) compare organ transplant recipients (OTRs) to non-transplanted cancer patients (No Tx) with respect to colon cancer characteristics (cancer location, tumor grade, number of lymph nodes examined) and treatment.

Characteristics **^a^**	Colon cancer			
	OTRs	No Tx	OR^2^ (95% CI)	p-value
	N (%)	N (%)		
Total	37 (100)	232 (100)		
Cancer location				0.002
Right colon	28 (76)	120 (52)	3.74 (1.59–8.84)	
Left colon	9 (24)	112 (48)	Base outcome	
Tumor grade				0.13
Low	24 (69)	179 (79)	Base outcome	
High	11 (31)	48 (21)	1.94 (0.82–4.62)	
*Missing*	*2*	*5*		
Perineural invasion				0.56
Yes	3 (10)	35 (18)	0.67 (0.18–2.55)	
No	26 (90)	161 (82)	Base outcome	
*Missing*	*8*	*36*		
Lymph nodes examined				0.58
0–11	6 (17)	25 (11)	1.37 (0.44–4.23)	
12–78	30 (83)	202 (89)	Base outcome	
*Missing*	*1*	*5*		
Emergency surgery				0.96
Planned	31 (84)	188 (81)	Base outcome	
Emergent	6 (16)	44 (19)	0.98 (0.36–2.63)	
Postoperative MDT meeting				0.10
Yes	27 (75)	203 (88)	0.34 (0.10–1.23)	
No	9 (25)	28 (12)	Base outcome	
*Missing*	*1*	*1*		
Neoadjuvant therapy^c^				-
Chemotherapy only	0 (0)	2 (1)	-	
No neoadjuvant therapy	32 (100)	197 (99)	Base outcome	
Adjuvant therapy^c^				0.02
Chemotherapy only	6 (19)	86 (43)	0.31 (0.11–0.85)	
No adjuvant therapy	26 (81)	113 (57)	Base outcome	
* Stage II cancer*				
* *Chemotherapy only	0 (0)	22 (22)	-	-
No adjuvant therapy	18 (100)	77 (78)	Base outcome	
* Stage III cancer*				0.18
* *Chemotherapy only	6 (43)	64 (64)	0.33 (0.07–1.67)	
* *No adjuvant therapy	8 (57)	36 (36)	Base outcome	

Abbreviations: OTR, organ transplant recipient. Tx, transplantation. OR, odds ratio; CI, confidence interval. N, number. MDT, multidisciplinary team.

^a^ Virtually all patients underwent microscopically radical surgery, and presented without tumor perforations, regardless of transplantation status; data not shown due to single observations.

^b^ All reported ORs refer to the contrast between OTRs, and non-transplanted cancer patients (No Tx), where the latter constitute the reference group. The regression models were adjusted for the matching factors sex, age at cancer diagnosis (±1 year), and year of cancer diagnosis, as well as region, cardiovascular comorbidity, attained level of education, and chronic dialysis at cancer diagnosis.

^c^ Among 32 OTRs and 199 non-transplanted patients with stage II-III cancer.

Among stage I-III rectal cancer patients treated with abdominal surgery, transplanted patients had seven times higher odds of having fewer (0–11 vs. 12–53) lymph nodes extracted and examined pathologically (OR:7.47, 95% CI:1.17–47.7), while no other differences in cancer characteristics and treatment were found (Supplementary Table S2). Adjuvant therapy was only administered to a few stage II-III patients and differences could therefore not be assessed.

In the subset of patients with stage IV CRC treated with abdominal surgery, and among patients who did not undergo potentially curative surgery, only descriptive results were presented due to generally low frequencies and high proportions of missing data (Supplementary Tables S3, S4). No stage IV OTR patients treated with abdominal surgery received neoadjuvant therapy (Supplementary Table S3).

### Other Comorbidity

As expected, all OTRs had CCI>0 at the time of cancer diagnosis, while 65% of the non-transplanted patients had CCI = 0 (Supplementary Table S5). In 33% of the transplanted CRC patients, and 11% of the non-transplanted, at least one other cancer diagnosis was recorded in the SCR before the CRC diagnosis. This difference was mainly explained by a higher frequency of non-melanoma skin cancer among the OTRs. Furthermore, 70 (71%) of the OTRs, and 134 (28%) of the non-transplanted patients, had been diagnosed with hypertension before CRC diagnosis.

### Survival and Relapse

**FIGURE 1:**
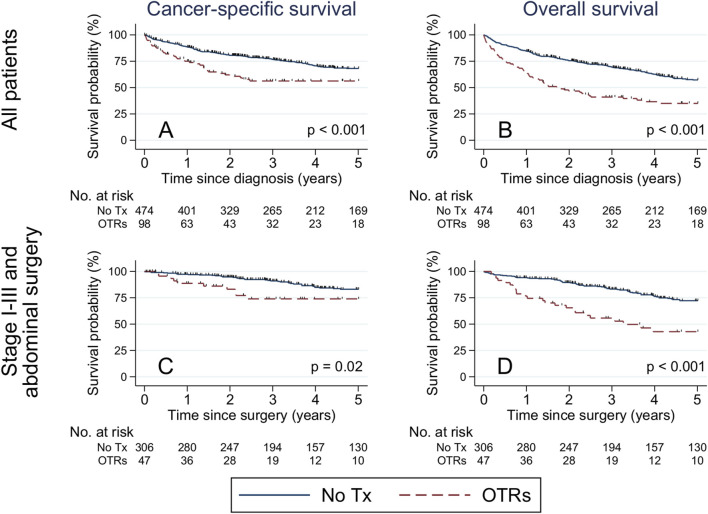
Kaplan-Meier estimates of cancer-specific and overall survival among all patients with colorectal cancer **(A,B)**, and restricted to stage I-III colorectal cancer patients treated with abdominal surgery **(C,D)**, stratified by medical history of solid organ transplantation. Differences were evaluated with the log-rank test. Abbreviations: No., number; Tx, transplantation; OTR, organ transplant recipient.

Overall, 240 patients (61 transplanted and 179 non-transplanted) died during 5 years of follow-up. Of those, 37 (61%) OTRs died of CRC, 5 (8%) of cardiovascular disease, 4 (7%) of infectious disease, 3 (5%) of diabetes, 3 (5%) of kidney disease, and 9 (15%) of other causes. Among non-transplanted patients 127 (71%) died of CRC, 8 (4%) of cardiovascular disease, 3 (2%) of infectious disease, 3 (2%) of lung disease, 3 (2%) of gastrointestinal disease, and 35 (20%) of other causes. The 5-year cancer-specific survival was 56% among transplanted patients, vs. 68% among non-transplanted patients (p_logrank_<0.001) (Figure 1A). The corresponding 5-year OS was 35% among transplanted patients, vs. 57% among non-transplanted patients (p_logrank_<0.001) (Figure 1B).

Among stage I-III CRC patients treated with abdominal surgery, 5-year cancer-specific survival was 74% among transplanted patients, vs. 83% among the non-transplanted (p_logrank_ = 0.02) (Figure 1C), while the corresponding 5-year OS was 43% among transplanted patients, vs. 72% among non-transplanted patients (p_logrank_<0.001) (Figure 1D). The 5-year cancer-specific mortality rate was two-fold increased (HR:2.16, 95% CI:1.48–3.15) among transplanted vs. non-transplanted patients overall, and 3.5-fold increased (HR: 3.50, 95% CI:1.64–7.43) among transplanted vs. non-transplanted stage I-III CRC patients treated with abdominal surgery (Table 4). Similarly, the overall mortality rate was two-fold increased (HR:2.11, 95% CI:1.55–2.88) among transplanted vs. non-transplanted CRC patients overall, and three-fold increased (HR:3.02, 95% CI 1.79–5.10) among transplanted vs. non-transplanted stage I-III CRC patients treated with abdominal surgery (Table 4). In a supplementary analysis with the date of administrative censoring set to 4th June 2022 (latest available date for vital status), 5-year OS proportions were 53% among OTRs and 79% among non-transplanted patients (p_logrank_ < 0.001), with a corresponding almost three-fold increased rate of death (HR:2.79, 95% CI:1.57–4.95) comparing OTRs to non-transplanted patients.

**TABLE 4:** Hazard ratios of cancer-specific death, overall death, and relapse, among stage I-III colorectal cancer patients treated with abdominal surgery, stratified by medical history of solid organ transplantation, with 5 years of follow-up. Administrative censoring occurred on Dec 31st, 2017, for cancer-specific and overall death, and on June 4th, 2022, for relapse.

Selection	Start of follow-up	Outcome	All colorectal cancer			Colon cancer			Rectal cancer		
			OTRs	No Tx	HR^a^ (95% CI)	OTRs	No Tx	HR^a^ (95% CI)	OTRs	No Tx	HR^a^ (95% CI)
			N (%)	N (%)		N (%)	N (%)		N (%)	N (%)	
All patients			98 (100)	474 (100)		73 (100)	352 (100)		25 (100)	122 (100)
	Date of diagnosis	Cancer-specific death	37 (38)	127 (27)	2.16 (1.48–3.15)	30 (41)	102 (29)	2.50 (1.64–3.81)	7 (28)	25 (20)	1.43 (0.58–3.52)
		Overall death	61 (62)	179 (38)	2.11 (1.55–2.88)	50 (68)	137 (39)	2.59 (1.83–3.66)	11 (44)	42 (34)	1.21 (0.59–2.48)
Stage I-III cancer and abdominal surgery **^b^**			47 (100)	306 (100)		37 (100)	232 (100)		10 (100)	74 (100)
	Date of surgery	Cancer-specific death	10 (21)	39 (13)	3.50 (1.64–7.43)	6 (16)	29 (13)	2.85 (1.14–7.12)	4 (40)	10 (14)	29.8 (3.77–235)
		Overall death	24 (51)	70 (23)	3.02 (1.79–5.10)	20 (54)	52 (22)	3.70 (2.07–6.62)	4 (40)	18 (24)	5.54 (1.26–24.4)
		Relapse	12 (26)	54 (18)	1.92 (0.98–3.76)	7 (19)	44 (19)	1.33 (0.57–3.12)	5 (50)	10 (14)	9.60 (1.84–50.1)

Abbreviations: OTR, organ transplant recipient; Tx, transplantation; HR, hazard ratio; CI, confidence interval; N, number.

^a^ All reported HRs refer to the contrast between OTRs, and non-transplanted cancer patients (No Tx), where the latter constitute the reference group. The regression models were adjusted for the matching factors cancer location (colon or rectum), sex, age at cancer diagnosis (±1 year), and year of cancer diagnosis, as well as region, cardiovascular comorbidity, attained level of education, and chronic dialysis at cancer diagnosis.

^b^ Subset of *All patients*.

Also among stage I-III CRC patients treated with abdominal surgery, 12 (26%) OTRs [7 (19%) with colon cancer and 5 (50%) with rectal cancer], and 54 (18%) non-transplanted patients [44 (19%) with colon cancer and 10 (14%) with rectal cancer], developed relapse within 5 years of surgery (Table 4). The relapse rate was similar among transplanted and non-transplanted colon cancer patients (HR:1.33, 95% CI:0.57–3.12), while transplanted rectal cancer patients had a ten times increased relapse rate (HR:9.60, 95% CI:1.84–50.1) compared with non-transplanted patients.

Only including kidney transplant recipients and their comparators in the survival analyses resulted in similar hazard ratios (data not shown). Furthermore, for overall survival, no significant interaction between transplantation status and colon cancer location was found.

## Discussion

In this nationwide, population-based study, we observed that transplanted stage I-III CRC patients were less likely than non-transplanted patients to undergo abdominal surgery and to receive adjuvant treatment for colon cancer, and more likely to develop relapse after rectal cancer. However, 90% of transplanted stage I-III CRC patients were treated with abdominal surgery, indicating that most transplanted CRC patients eligible for abdominal surgery actually receive such treatment. The 5-year cancer-specific survival was lower among transplanted compared with non-transplanted patients, both among all patients and among stage I-III CRC patients treated with abdominal surgery. This indicates that the lower cancer-specific survival seen among OTRs could to some extent be due to differences in the likelihood of receiving surgical, adjuvant, and potentially also neoadjuvant, treatment. Nevertheless, stage I-III colon cancer patients treated with abdominal surgery experienced long-term cancer-specific survival that was comparable to non-transplanted individuals, which could indicate that aggressive cancer treatment is justified among OTRs.

While several case reports/series of CRC among OTRs have been published previously, comparative studies of consecutive patients are few. Papaconstantinou et al demonstrated worse OS both for localized and regionally metastasized cancer, but not for cancers with distant metastases, among 150 OTRs with CRC compared with CRC patients in the general population [15]. Kim JY et al compared 17 post-transplant CRC patients to 170 non-transplanted CRC patients matched on closest date of surgery. As in our study, they found no difference in stage and tumor histology, but reported less extensive lymph node dissection and lower frequency of administration of adjuvant therapy (50% vs. 86%) among OTRs than non-transplanted patients, although no formal analyses or adjustments could be made [16]. Among patients with CRC stage III-IV, the 5-year OS was lower among OTRs. Khoury and colleagues demonstrated lower OS and disease-free survival among immunosuppressed CRC patients compared with immunocompetent patients, and argued that the differential DFS was likely due to increased risk of distant recurrence [17]. Merchea et al reported that OTRs were prone to developing right-sided colon cancer (70%) and present with stage IV disease (25%-30%), with a 5-year OS of 42.5% [18, 19]. In our study, generally higher age at diagnosis might partly explain the higher proportion of stage IV disease (40% for colon cancer) and lower 5-year OS (35%). Finally, Kim M et al matched 33 kidney and liver transplant recipients with CRC to non-transplanted surgically treated CRC patients [20]. Adjuvant chemotherapy proportions (52% of OTRs, and 61% of non-transplanted patients) and 5-year OS (80% among OTRs) were similar.

End-stage organ failure leading to organ transplantation always represents significant comorbidity, which, depending on recipient age, accumulated comorbid conditions, and overall clinical status, has implications for both surgical and neo-/adjuvant cancer treatment. However, comorbidity can also be dramatically improved post-transplant, which is difficult to classify with, e.g., CCI. At cancer diagnosis, the risk of damaging a kidney transplant correlates with (worsening) transplant function, which will influence treatment decisions especially when the expected beneficial effect of chemo-/radiotherapy is moderate. Nevertheless, in our study, adjuvant colon cancer treatment was administered to 0/18 transplanted stage II patients, compared with 22/99 non-transplanted, begging the question whether some OTRs with stage II colon cancer might have been eligible for adjuvant treatment. The low frequency of adjuvant chemotherapy administered to both transplanted and non-transplanted rectal cancer patients is expected, as the benefit of treatment with chemotherapy is lower for rectal cancer than for colon cancer, while such treatment entails substantial risk of morbidity and adverse reactions [10]. Furthermore, radiotherapy may have been deemed contraindicated to a larger extent among transplanted rectal cancer patients, due to possibly harmful effects to the transplanted ureter. However, modern, more precise radiotherapy methods should in many cases enable neoadjuvant radiation for rectal cancer.

Similar to other studies, we found an overrepresentation of right-sided colon cancers among transplanted compared to non-transplanted CRC patients [16, 21]. Right-sided colon cancers have been associated with worse prognosis, and may overall respond to a lower degree to chemotherapy than left-sided; however, this did not impact treatment decisions in the present study [22, 23].

We suggest that MDT meetings should be held whenever an OTR develops a *de novo* cancer where adjuvant treatment would normally be recommended in addition to surgery. Preferably, these MDT meetings would involve not only, e.g., cancer surgeons and oncologists, but also organ transplant specialists, to prevent both over- and undertreating of the cancer. Hellström et al showed that MDT meetings including kidney transplant consultants altered the initial oncological treatment for post-transplant solid or hematological cancer in 52% of the patients, after which 82% were treated in accordance with national guidelines [24]. However, the extra resource allocation required implicates that such conferences need further scientific evaluation.

While strengths of this study include its population-based nature, usage of registers with excellent coverage, and a comparatively large cohort, we recognize that certain subgroup comparisons should be interpreted within the limitations of available clinical data and statistical power reflected in wide confidence intervals for some contrasts. Information on transplant function and clinical status at cancer diagnosis was available only through proxies of register records of chronic dialysis and cardiovascular comorbidity, and may have been underestimated to some extent. We further adjusted for healthcare region and socioeconomic status through attained education level. We cannot exclude residual confounding by center/surgeon effects although recent Nordic studies on associations between annual hospital volume of CRC surgery and outcome have found either no beneficial effects of centralization, or a small benefit overall that might be confined to certain patient subgroups [25–28]. It was also unclear whether transplantation specialists were involved in the oncological decision-making. Detailed oncological treatment data were not registered and some tumor-specific risk factors, e.g., presence of tumor deposits, were only registered during part of the study period, which limited the ability to fully assess given treatments. Even though we have adjusted for cardiovascular comorbidity and dialysis in our analyses, it might also be argued that transplantation is merely a proxy for comorbidity, which could explain the observed treatment differences. This does not, however, alter our conclusion that OTRs are treated differently, and we have here reported the extent of these differences, which can form the basis for future studies.

## Conclusion

While most transplanted CRC patients with stage I-III cancer underwent abdominal surgery for their cancer, they were still less likely to undergo abdominal surgery than non-transplanted patients. Furthermore, among stage I-III CRC patients treated with abdominal surgery, transplanted colon cancer patients were less likely to be treated with adjuvant chemotherapy, and transplanted rectal cancer patients had higher rate of relapse, than non-transplanted patients with the same cancer type. Also, transplantation was associated with worse cancer-specific and overall survival among CRC patients. We suggest that MDT meetings, involving organ transplant specialists, should be considered for all post-transplant cancer patients to ensure optimal treatment in the post-transplant setting.

## Data Availability

The datasets presented in this article are not readily available because they are based on detailed pseudonymized health data, the use of which is guided by ethical approval to the principal investigator. Data can only be shared based on additional evaluation and approvals by regulatory authorities in Sweden. Requests to access the datasets should be directed to the corresponding author.

## Abbreviations

CCI, Charlson comorbidity index; CI, Confidence interval; CRC, Colorectal cancer; HR, Hazard ratio; MDT, Multidisciplinary team; NPR, National Patient Register; OR, Odds ratio; OS, Overall survival; OTR, Organ transplant recipient; RRR, Relative risk ratio; SCR, Swedish Cancer Register; TNM, Tumor-node-metastasis.
