# Supplementary material for: Previous Solid Organ Transplantation Influences Both Cancer Treatment and Survival Among Colorectal Cancer Patients
Source: Transpl Int. 2024 Sep 20;37:13173. doi: 10.3389/ti.2024.13173 (PMC11449720; doi:10.3389/ti.2024.13173)
Supplement: Supplementary file 1 [file DataSheet1.pdf]

SUPPLEMENTARY MATERIAL

Table S1. Surgery codes used for identifying solid organ transplantation in medical history prior to colorectal cancer diagnosis.

Table S2. Characteristics of stage I-III rectal cancer patients treated with abdominal surgery, by medical history of solid organ transplantation. The odds ratios (OR) compare organ transplant recipients (OTRs) to non-transplanted cancer patients (No Tx) with respect to rectal cancer characteristics (location, tumor grade, number of lymph nodes examined) and treatment.

Table S3. Characteristics of stage IV colorectal cancer patients treated with abdominal surgery, stratified by medical history of solid organ transplantation.

Table S4. Characteristics of colorectal cancer patients not treated with potentially curative surgery such as either abdominal surgery or endoscopic polypectomy (i.e., patients deemed non-operable, and patients with distant metastases), by medical history of solid organ transplantation.

Table S5. Charlson Comorbidity Index (CCI) score distribution among colorectal cancer patients, by medical history of solid organ transplantation.

Table S1. Surgery codes used for identifying solid organ transplantation in medical history prior to colorectal cancer diagnosis.

Type of transplant	Codes used
Kidney	KAS10, KAS20, 6070
Liver	JJC00, JJC10, JJC20, JJC30, JJC40, 5200, 5202
Pancreas	JLE00, JLE03, JLE10, JLE16, JLE20, JLE30, 5530, 5531
Heart	FQA00, FQA10, FQA20, FQA30, FQA40, FQA96, 3085
Heart and lung	FQB00, FQB10, FQB20, FQB30, FQB96, 6085
Lung	GDG00, GDG03, GDG10, GDG13, GDG30, GDG96, 3590
Bowel	JFE00

Table S2. Characteristics of stage I-III rectal cancer patients treated with abdominal surgery, by medical history of solid organ transplantation. The odds ratios (OR) compare organ transplant recipients (OTRs) to non-transplanted cancer patients (No Tx) with respect to rectal cancer characteristics (location, tumor grade, number of lymph nodes examined) and treatment.

**Characteristics**[1]	**Rectal cancer**			
	**OTRs**	**No Tx**	**OR**[2] **(95% CI)**	**p-value**
	N (%)	N (%)		
**Total**	10 (100)	74 (100)		
**Cancer location (cm from anal verge)**				0.30
<6	4 (40)	21 (29)	Base outcome	
6-15	6 (60)	52 (71)	0.39 (0.07-2.29)	
Missing	*0*	*1*		
**Tumor grade**				0.48
Low	7 (78)	51 (84)	Base outcome	
High	2 (22)	10 (16)	2.35 (0.22-25.0)	
Missing	*1*	*13*		
**Perineural invasion**				-
Yes	0 (0)	6 (12)	-	
No	8 (100)	46 (88)	Base outcome	
Missing	*2*	*22*		
**Lymph nodes examined**				0.03
0-11	6 (60)	22 (29)	7.47 (1.17-47.7)	
12-53	4 (40)	51 (71)	Base outcome	
Missing	*0*	*1*		
**Neoadjuvant (chemo)radiotherapy**				0.45
Yes[3]	4 (40)	53 (72)	0.55 (0.11-2.66)	
No	6 (60)	21 (28)	Base outcome	
**Postoperative MDT meeting**				-
Yes	8 (100)	52 (88)	-	
No	0 (0)	7 (12)	Base outcome	
Missing	*2*	*15*		

Abbreviations: OTR, organ transplant recipient. Tx, transplantation. OR, odds ratio. CI, confidence interval. N, number. MDT, multidisciplinary team.

[1] Virtually all patients underwent microscopically radical surgery, and presented without tumor perforations, regardless of transplantation status; data not shown due to single observations. Data regarding adjuvant therapy, and stage-specific data regarding neoadjuvant (chemo)radiotherapy, also not shown due to single observations.

[2] All reported ORs refer to the contrast between OTRs and non-transplanted cancer patients (No Tx), where the latter constitute the reference group. The regression models were adjusted for the matching factors sex, age at diagnosis (±1 year), and year of cancer diagnosis, as well as region, cardiovascular comorbidity, attained level of education, and chronic dialysis at cancer diagnosis.

[3] Chemotherapy was administered to 0/4 transplanted patients, and 12/53 non-transplanted, in addition to radiotherapy. The frequency of neoadjuvant (chemo)radiotherapy administered was accordant with Swedish treatment practices (see reference no. 7 in the main article).

Table S3. Characteristics of stage IV colorectal cancer patients treated with abdominal surgery, stratified by medical history of solid organ transplantation.

Characteristics[1]	Colon cancer		Rectal cancer	
	**OTRs**	**No Tx**	**OTRs**	**No Tx**
	N (%)	N (%)	N (%)	N (%)
**Total**	9 (100)	57 (100)	4 (100)	8 (100)
**Metastases at diagnosis**				
Liver	4 (44)	26 (46)	2 (50)	5 (63)
Lung or other	4 (44)	33 (58)	0 (0)	2 (25)
**Tumor grade**				
High	4 (57)	17 (34)	0 (0)	2 (29)
Low	3 (43)	33 (66)	2 (100)	5 (71)
Missing	*2*	*7*	*2*	*1*
**Neoadjuvant therapy**				
Any neoadjuvant therapy	0 (0)	6 (11)	0 (0)	4 (62)
No neoadjuvant therapy	9 (100)	51 (89)	4 (100)	3 (38)
Missing	*0*	*0*	*0*	*1*

Abbreviations: OTR, organ transplant recipient. Tx, transplantation. N, number.
[1] Data regarding adjuvant therapy not shown due to single observations.

Table S4. Characteristics of colorectal cancer patients not treated with potentially curative surgery such as either abdominal surgery or endoscopic polypectomy (i.e., patients deemed non-operable, and patients with distant metastases), by medical history of solid organ transplantation.

**Characteristics**	**Colorectal cancer**	
	**OTRs**	**No Tx**
	N (%)	N (%)
**Total**	26 (100)	56 (100)
**cTNM**		
**cT**		
cT1-3	7 (27)	18 (32)
cT4	7 (27)	11 (20)
cTx	12 (46)	27 (48)
**cN**		
cN0	6 (23)	7 (13)
cN1-2	6 (23)	21 (38)
cNx	14 (54)	28 (50)
**cM**		
cM0	8 (31)	11 (20)
cM1	16 (62)	43 (77)
cMx	2 (8)	2 (4)
**Metastases (if cM1)**		
Liver	15 (94)	39 (91)
Lung or other	5 (31)	28 (65)
**Planned palliative treatment**		
Yes	6 (50)	27 (71)
No	6 (50)	11 (29)
Missing	*14*	*18*

Abbreviations: OTR, organ transplant recipient. Tx, transplantation. N, number. cTNM, clinical tumor-node-metastasis stage.

Table S5. Charlson Comorbidity Index (CCI) score distribution among colorectal cancer patients, by medical history of solid organ transplantation.

Characteristics					
	**No Tx**	**OTRs**			**All patients**
		**Any Tx**	**Kidney Tx**	**Other[1] Tx**	
	N (%)	N (%)	N (%)	N (%)	N (%)
**Total**	474 (100)	98 (100)	75 (100)	23 (100)	572 (100)
**Type of comorbidity (weighted CCI score)**					
No comorbidity registered	306 (65)	0 (0)	0 (0)	0 (0)	318 (54)
Cerebrovascular disease (1)	25 (5)	9 (9)	9 (12)	0 (0)	34 (6)
Chronic obstructive pulmonary disease (1)	15 (3)	7 (7)	4 (5)	3 (13)	22 (4)
Congestive heart failure (1)	24 (5)	10 (10)	8 (11)	2 (9)	34 (6)
Dementia (1)	0 (0)	0 (0)	0 (0)	0 (0)	0 (0)
Diabetes (1)	0 (0)	0 (0)	0 (0)	0 (0)	0 (0)
Mild/severe liver disease (1/3)	2 (0)	15 (15)	5 (7)	10 (43)	17 (3)
Myocardial infarction (1)	21 (4)	14 (14)	11 (15)	3 (13)	35 (6)
Other chronic pulmonary disease (1)	11 (2)	2 (2)	2 (3)	0 (0)	13 (2)
Peptic ulcer (1)	8 (2)	7 (7)	5 (7)	2 (9)	15 (3)
Peripheral vascular disease (1)	12 (3)	7 (7)	5 (7)	2 (9)	19 (3)
Rheumatic disease (1)	13 (3)	7 (7)	7 (9)	0 (0)	20 (4)
Diabetes with end organ damage (2)	19 (4)	15 (15)	12 (16)	3 (13)	34 (6)
Hemiplegia (2)	2 (0)	2 (2)	2 (3)	0 (0)	4 (1)
Malignancy with or without metastases (2/6)	52 (11)	32 (33)[2]	27 (36)	5 (22)	84 (15)
Severe kidney disease (2)	7 (1)	83 (85)	73 (97)	10 (43)	90 (16)
Acquired immuno-deficiency syndrome (6)	7 (2)	2 (2)	0 (0)	2 (9)	9 (2)
**Sums of weighted CCI scores**					
0	306 (65)	0 (0)			
1	47 (10)	5 (5)			
2	69 (15)	23 (23)			
3-4	31 (7)	37 (38)			
5-6	13 (3)	23 (23)			
7-10	8 (2)	10 (10)			

Abbreviations: OTR, organ transplant recipient. Tx, transplantation. CCI, Charlson comorbidity index.

[1] Liver, pancreas and kidney, heart, or lung transplantation.

[2] The overrepresentation of cancer history in transplanted patients was mainly driven by non-melanoma skin cancer.
